# Quorum signaling and sensing by nontypeable *Haemophilus influenzae*

**DOI:** 10.3389/fcimb.2012.00100

**Published:** 2012-07-19

**Authors:** W. Edward Swords

**Affiliations:** Department of Microbiology and Immunology, Wake Forest University Health SciencesWinston-Salem, NC, USA

**Keywords:** biofilm, *Haemophilus influenzae*, otitis media, quorum signals, chronic

## Abstract

Quorum signals are diffusible factors produced by bacteria that coordinate communal responses. For nontypeable *Haemophilus influenzae* (NT*Hi*), a series of recent papers indicate that production and sensing of quorum signals are determinants of biofilm formation/maturation and persistence *in vivo*. In this mini-review I will summarize the current knowledge about quorum signaling/sensing by this organism, and identify specific topics for additional study.

## Introduction

Bacterial quorum sensing involves community-dependent regulation of bacterial gene expression by means of soluble signal molecules that are released in a density-dependent manner (Miller and Bassler, 2001; Henke and Bassler, 2004; Waters and Bassler, 2005). These signaling molecules are chemically diverse, and include the so-called competence factor peptides of pneumocci and other gram-positive bacteria, homoserine lactones, and derivatives of the bacterial metabolic by-product dihydroxypentanedione (DPD), which is also referred to as autoinducer-2 (AI-2). Because production of DPD is widely conserved among bacterial species, it has been referred to as an interspecies quorum signal (Surette et al., 1999).

Quorum sensing has been shown to coordinate group activities among bacterial populations, including formation and maturation of biofilm communities (Parsek and Singh, 2003; Henke and Bassler, 2004; Irie and Parsek, 2008; Shrout et al., 2011). In this mini-review, I discuss the current knowledge about quorum signaling and sensing in nontypeable *Haemophilus influenzae* (NT*Hi*), and highlight potential areas for future study.

### Relationship between DPD (AI-2) quorum signals and virulence

The current published work concerning NT*Hi* quorum signaling and sensing is summarized in Table 1. Quorum signaling for this species was first suggested by presence of the *luxS* genetic determinant of DPD production in the *H. influenzae* Rd genomic sequence (Surette et al., 1999). Later, Daines and colleagues showed that numerous NT*Hi* strains produced quorum signal as detected by the *Vibrio harveyi* bioluminescence assay (Daines et al., 2005). Also in this study, *luxS* transcript levels were shown to be increased during infection of epithelial cells, and mutants lacking *luxS* were also shown to retain the capacity to form biofilms, have increased invasion of epithelial cells, and cause more severe otitis media disease in the chinchilla infection model.

**Table 1:** **Current knowledge about quorum signaling/sensing in *H. influenzae***.

**Finding**	**Reference**
NT*Hi luxS* mutants have increased invasion	Daines et al., 2005
NT*Hi luxS* mutants cause acute otitis media with greater inflammation	Daines et al., 2005
Quorum signaling promotes NT*Hi* biofilm maturation	Armbruster et al., 2009
Quorum signaling affects lipooligosaccharide composition	Armbruster et al., 2009
NT*Hi* quorum signals affect *M. catarrhalis*	Armbruster et al., 2010
RbsB is a determinant of quorum signal uptake for strain NT*Hi* 86-028NP	Armbruster et al., 2011

Later work from our laboratory expanded on this work to show that while isogenic NT*Hi* mutants lacking *luxS* do retain the capacity to form biofilms, quantitative assessment of the biofilm structure by confocal microscopic analysis showed significantly reduced thickness and density, which was restored by complementation or by co-culture with the parental strains (Armbruster et al., 2009). Chinchilla infection studies also revealed that *luxS* mutants caused a more acute, inflammatory infection and that long-term persistence of *luxS* mutants was significantly reduced as compared with the parental strain (Armbruster et al., 2009). These changes in biofilm were correlated with shifts in the lipooligosaccharide glycolipids on the bacterial surface, which had previously been shown by our group to promote biofilm maturation and persistence *in vivo* (Hong et al., 2007a,b).

More recently, the RbsB protein was shown to mediate uptake of DPD quorum signals for NT*Hi* 86-028NP (Armbruster et al., 2011). RbsB is a periplasmic binding protein which functions as part of an ABC transporter for ribose sugars (Park et al., 1999), and has been shown to function in AI-2 uptake in other bacterial species (Shao et al., 2007). Similar to *luxS* mutants, isogenic *rbsB* mutants were demonstrated to produce biofilms with significantly reduced thickness and density as compared to the parental NT*Hi* strain. These changes in biofilm were correlated with changes in the lipooligosaccharide content and a persistence defect in the chinchilla infection model (Armbruster et al., 2011). However, given the genomic diversity among NT*Hi* strains, it is important to note that there is a strong possibility for other determinants of quorum signal uptake (or absence of RbsB) in other strains (Pereira et al., 2009). In support of this idea, examination of the 18 NT*Hi* publicly accessible genomic sequences reveals that while the majority of strains (12/18) would be predicted to only have the Rbs system for uptake, orthologs of the Lsr system associated with quorum signal uptake and sensing in other bacterial species are found in 3/18 strains; in one of these the Rbs transporter is not found. Notably, in 3/18 strains there were no predicted sequences for either transporter. Thus, the potential exists for significant mechanistic diversity in quorum signal uptake (and presumably, sensing) among different NT*Hi* strains. We are currently addressing this important topic.

### Interspecies quorum signaling

In addition to the impact of quorum signaling on NT*Hi* biofilms, recent work has demonstrated that NT*Hi* quorum signals may impact *Moraxella catarrhalis*, an opportunistic pathogen that inhabits many of the same host environments within the airway. Growth of *M. catarrhalis* within a polymicrobial biofilm with nontypeable *H. influenzae* was shown to promote antibiotic resistance and persistence within the chinchilla infection model (Armbruster et al., 2010). In support of quorum signaling as a mechanism for these effects on *M. catarrhalis*, there was no significant benefit in terms of antibiotic resistance or persistence *in vivo* in parallel experiments using an isogenic NT*Hi luxS* mutant strain. Notably, no AI-2 quorum signal production was detected for any *M. catarrhalis* strain, and recent data analyzing a number of sequenced *M. catarrhalis* genomes show that none have a homologue for the *luxS* genetic determinant of this quorum signal (Davie et al., 2011). Moreover, *M. catarrhalis* bacteria had the capacity to take up purified DPD from culture supernatants, and addition of purified DPD to *M. catarrhalis* bacteria also promoted biofilm density and antibiotic resistance *in vivo* (Armbruster et al., 2010). Taken together, these results show that *M. catarrhalis* “eavesdrops” on NT*Hi* quorum signals to coordinate its biofilm development. These results are consistent with epidemiologic data that indicate a significant correlation of *M. catarrhalis* with NT*Hi* co-infection in clinical samples from patients with otitis media or other opportunistic airway infections (Pettigrew et al., 2008; Verhaegh et al., 2011).

### Controversies, remaining questions and topics for additional study

**Figure 1:**
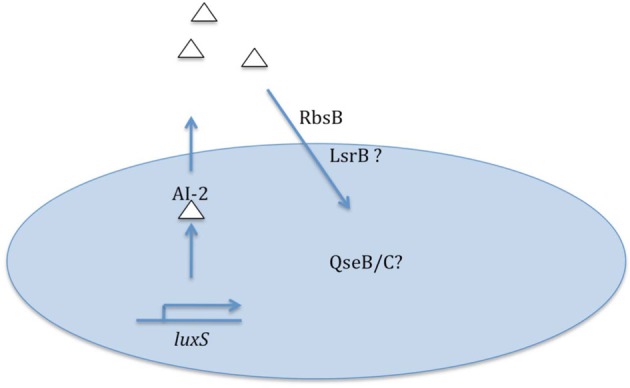
**Summary of data and some remaining questions regarding nontypeable *H. influenzae* quorum signaling/sensing**.

While present data clearly establish the importance of quorum signaling/sensing for some NT*Hi* model strains, much remains to be learned on this topic and its relationship to virulence (summarized in Figure 1). The magnitude and kinetics of quorum signal production by different NT*Hi* strains is not presently known, and there is a distinct possibility that some strains may not fit with the current knowledge that has mostly been derived from NT*Hi* 86-028NP. As highlighted above, genomic analyses clearly indicate presence of other potential AI-2 transporters in some NT*Hi* genomes; whether these strains have greater capacity for signal uptake (and presumably sensing) is a subject for additional study. All sequenced NT*Hi* strains have homologs to the QseB/C two-component signaling system that mediates sensing of AI-2/DPD signals for some other bacterial species; the role of these factors in sensing of quorum signal by NT*Hi* is not presently clear. Finally, generation of NT*Hi* mutant strains in which AI-2 quorum signal may be artificially induced would be of great help not only in clarifying the direct linkage of quorum signal to biofilm formation/maturation but also in defining the consequences of quorum sensing for the bacterial population.

It should also be pointed out that the relevance of biofilms for NT*Hi* infection is not universally accepted (Moxon et al., 2008). Criticisms of this concept have included the lack of a defined matrix component expressed exclusively during biofilm growth, observation of aggregated bacteria with some of the descriptive qualities associated with biofilm in plate cultures, and lack of evidence for a controlled development of a differentiated community within NT*Hi* biofilms/aggregates. Certainly, there was at the time of this commentary a great need for better understanding of the process of biofilm development and the role(s) of biofilms in the persistence of NT*Hi* bacteria *in vivo*. For example, prior to our demonstration of NT*Hi* survival within neutrophil extracellular traps (NET; Hong et al., 2009; Juneau et al., 2011), it could have been plausible to consider the possibility that the surface-adherent bacteria, rather than persisting, were being killed. Likewise, the findings discussed above regarding role(s) for synthesis and uptake of quorum signal (Armbruster et al., 2009, 2011), and restoration of biofilm phenotype by addition of culture supernatants or synthetic DPD to *luxS* mutants (Armbruster et al., 2009, 2011), provide additional evidence for coordinated development of a biofilm. It is also important to note work from other species indicating roles for nutrient composition of growth media in biofilm formation/maturation that can equal or even surpass that of quorum signaling/sensing (Shrout et al., 2006, 2011). This will surely be an important variable for additional study with regard to NT*Hi* biofilms.

### Conflict of interest statement

The author declares that the research was conducted in the absence of any commercial or financial relationships that could be construed as a potential conflict of interest.
